# Bone mineral density in egyptian children with juvenile idiopathic arthritis: possible correlation to serum RANKL / osteoprotegerin (OPG) ratio and OPG gene polymorphisms

**DOI:** 10.1186/s12969-023-00843-6

**Published:** 2023-06-16

**Authors:** Riham Eid, Maha Abdelsalam, Aya Ahmed Fathy, Hadil M. Abolenein, Eman Bakr Elmarghany, Aya Ahmed El-Hanafy, Nashwa Hamdy, Dina Salama Abd-Elmagid, Nermeen A. Niazy, Dina M. Abd-El Ghaffar

**Affiliations:** 1 Paediatric Nephrology Unit, Faculty of Medicine, Mansoura University Children’s Hospital, Mansoura University, Mansoura, 35561 Egypt; 2 Immunology Unit, Clinical Pathology Department, Faculty of Medicine, Mansoura University, Mansoura, Egypt; 3 Department of Immunology, Egypt Center for Research and Regenerative Medicine (ECRRM), Cairo, 11517 Egypt; 4 Public health and Community Department, Faculty of Medicine, Mansoura University, Mansoura, Egypt; 5 Paediatric Endocrinology and Diabetes Unit, Faculty of Medicine, Mansoura University Children’s Hospital, Mansoura University, Mansoura, Egypt; 6 Rheumatology, Rehabilitation and Physical Medicine Department, Faculty of Medicine, Mansoura University, Mansoura, Egypt; 7 Medical Biochemistry Department, Faculty of Medicine, Mansoura University, Mansoura, Egypt; 8 Paediatric Neurology Unit, Faculty of Medicine, Mansoura University Children’s Hospital, Mansoura University, Mansoura, Egypt

**Keywords:** sRANKL, Osteoprotegerin, OPG gene, Juvenile idiopathic arthritis, Bone mineral density

## Abstract

**Background:**

Children with juvenile idiopathic arthritis (JIA) are at higher risk of decreased bone mineral density (BMD) compared with healthy children due to genetic, disease and medication-related causes. This study aims to investigate the possible effects of osteoprotegerin (OPG) gene polymorphisms and serum levels of osteoprotegerin (OPG) and receptor activator of nuclear factor κB-ligand (RANKL) and RANKL/OPG ratio on BMD in children with JIA.

**Methods:**

OPG gene rs2073617, rs3134069, serum RANKL, OPG and RANKL/OPG ratio were evaluated in 60 JIA children and 100 matched healthy controls. BMD was evaluated by lumbar dual energy X-ray absorptiometry (DEXA) according to which patients were classified in 2 groups (DEXA z-score above and below − 2). Composite disease activity was measured using the Juvenile Arthritis Disease Activity Score (JADAS) 27-joints. Articular damage was scored using the juvenile arthritis damage index (JADI).

**Results:**

Patients aged 12.05 ± 3.2 years, included 38 females and 31% had BMD z-score below-2. Systemic-onset JIA was the most frequent phenotype (38%). Genotypes and alleles frequencies of the 2 studied polymorphisms did not differ between patients and controls (*p* > 0.05 for all) while serum RANKL and RANKL/OPG ratio were significantly higher in patients compared to controls (*p* = < 0.001 and 0.03 respectively). Patients with BMD < -2 had significantly greater frequencies of rs2073617 TT genotype and T allele (*p* < 0.001), higher serum RANKL, RANKL/OPG ratio (*p* = 0.01, 0.002), female predominance (*p* = 0.02), higher articular and extra-articular damage index (*p* = 0.008,0.009) and more frequent steroid usage (*p* = 0.02) compared to patients with BMD z-score >-2. Multivariate analysis showed rs2073617 TT genotype, RANKL/OPG ratio, long disease duration (above 36 months) and use of steroid to be associated with decreased BMD (*p* = 0.03,0.04,0.01,0.01 respectively) in JIA children.

**Conclusions:**

Egyptian children with JIA have decreased BMD. rs2073617 TT genotype and T allele, RANKL/OPG ratio are possible determinants of reduced BMD in JIA. Our results underline the importance of frequent monitoring of BMD in JIA children and trying to control disease activity to preserve long term bone health.

**Supplementary Information:**

The online version contains supplementary material available at 10.1186/s12969-023-00843-6.

## Background

Children with juvenile idiopathic arthritis (JIA) are at increased risk of reduced bone mineral density (BMD) which is related to diverse factors as chronic inflammation, medications, nutrition, and decreased physical activity. It is important to detect the early changes in BMD in JIA to identify patients at risk to develop reduced bone mass [1]. Dual energy X-ray absorptiometry (DEXA) is the most frequently used method for evaluating the bone mineralization status in different ages [2] but it is technically difficult to perform and to interpret its results in children with growth retardation and hormonal disturbances. So, new more applicable markers are required [3].

Tumour necrosis factor (TNF) is considered the chief inflammatory pathogenic mediator for chronic arthritis [4]. Among the TNF family, the receptor activator of nuclear factor-κB (RANK),RANK ligand (RANKL), and osteoprotegerin (OPG) are involved in many immunological and skeletal diseases characterized by bone resorption including inflammatory arthritis[5]. Expression of the RANKL by osteoblasts is crucial to osteoclastogenesis. OPG is a soluble receptor for RANKL that prevents RANK/RANKL interaction [6, 7]. Therefore, the RANKL/OPG ratio is critical for guiding bone resorption. Altered RANKL/ OPG ratio has been described in multiple autoimmune diseases and has been linked to decreased BMD [8, 9]. As genetic background has a pivotal role in determining peak bone mass [10], and OPG protect from bone resorption and cartilage damage [11, 12], the OPG gene may be a good contestant to identify subjects with high risk of compromised BMD.

Understanding the impact of JIA on children’s bone health may help enhanced prevention and treatment of these complications in children. In the current work, we aimed to assess the RANKL/OPG balance in JIA patients compared with healthy controls and correlate serum levels of RANKL, OPG and RANKL/OPG ratio with clinical phenotypes, BMD, disease activity and damage indices. In addition, 2 polymorphisms of OPG gene (rs2073617 and rs3134069) have been studied and correlated to BMD. To the best of our knowledge these 2 polymorphisms were not studied before in JIA despite their reported role in impaired BMD in Egyptian childhood systemic lupus erythematosus (SLE) [13], and in adults with rheumatoid arthritis (RA) [14].

## Methods

### Study participants

Sixty patients with JIA diagnosed according to the International League of Associations for Rheumatology (ILAR) criteria [15] were recruited from paediatric Rheumatology clinic, Mansoura University Children’s Hospital. One hundred age and sex matched children, apparently healthy and with no features suggestive or family history of rheumatic disorders were recruited as controls (all control group have been included before in previous research [13]**).**

All patients had normal renal function (at time of sampling) defined as normal estimated glomerular filtration rate (eGFR) for their age (as renal clearance plays an important role in the excretion of OPG). Exclusion criteria included patients with reduced eGFR and patients with associated autoimmune disorders or mixed connective-tissue diseases and other disorders affecting bone status as rickets, thyroid and parathyroids disorders.

For follow up of our patients; when the criteria for inactive disease [16, 17] are met for a minimum of six consecutive months while the patient is receiving anti-rheumatic medications, the patient is classified as being in the state of clinical remission with medication. When the criteria for inactive disease are met for a minimum of 12 consecutive months after the patient has discontinued all anti-rheumatic medications, the patient is classified as being in the state of clinical remission without medication. However, as we did not follow up our patients for the required period, Composite disease activity was measured (at time of sampling) by the Juvenile Arthritis Disease Activity Score (JADAS) 27-joints (range 0–57) [18]. Long term damage (articular and extra-articular) was scored using the Juvenile Arthritis Damage Index (JADI) [19].

None of the included 60 patients received bisphosphonates up to the time of sampling and BMD assessment in the study. Unfortunately, Data regarding calcium and vitamin D consumption were not included in the analysis because of patients’ inconstant interrupted undocumented doses and intake durations.

### Sample collection and serum RANKL /OPG measurement

A total of 5ml blood samples were collected from all subjects by venepuncture. Two ml of blood were added to Ethylene Di-amine Tetra-acetic Acid (EDTA) containing tube and stored at -20 °C until extraction of DNA. The remaining three ml blood were delivered in a dry tube, allowed to clot at room temperature for about half an hour and then centrifuged at 3000 round per minute (rpm) for 15 min to obtain the serum. The serum was stored at − 80 °C until further use by enzyme-linked immune sorbent assay (ELISA) technique. Both protocols were performed according to the manufacturer’s instructions. The unit of the standards in the two ELISA kits used in the study was ng per L. The RANKL/OPG ratio was calculated and included in the statistical analysis.

### Single nucleotide polymorphism genotyping

For the control group, we used the results of genotyping of the 100 controls included in our previous research [13]. While for cases, DNA was extracted from peripheral blood samples using Quick-DNA (Zymo research, Orange, CA, USA, Cat. No D3024) according to standard procedures and screened for the T950C and T245G, OPG gene polymorphisms using polymerase chain reaction (PCR)-based restriction fragment length polymorphism technique.

### Rs2073617

**Fig. 1:**
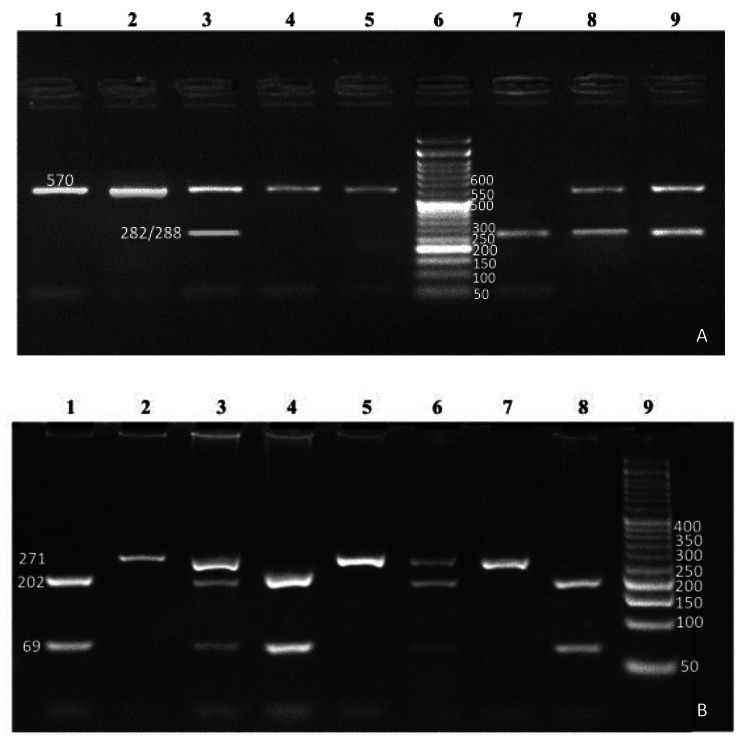
(**A**) Electrophoresis pattern of T950C (rs2073617) polymorphism resolved on 2.5% agarose gel. Lane 1, 2, 4, 5: TT homozygote (570 bp); Lane 3, 8, 9: TC heterozygote (570, 282 and 288 bp); lane 7: CC homozygote (288 bp and 282 bp fragments ”appear as one band”); Lane 6: 50 bp DNA ladder. (**B**) Electrophoresis pattern of T245G (rs3134069) polymorphism resolved on 3% agarose gel. Lane 2, 5, 7: GG homozygote (271 bp); Lane 1, 4, 8: TT homozygote (202 bp and 69 bp fragments); lane 3, 6: TG heterozygote (271, 202 and 69 bp); Lane 9: 50 bp DNA ladder

A 570-bp fragment around the T950C polymorphism (rs2073617) was amplified as described by Soufi et al. [20]. The PCR products were digested with HincII restriction endonuclease (Fermentas, Burlington, ON, Canada) and digestion products were separated on 2.5% agarose gels. In the presence of a C nucleotide at position 950, the 570 bp PCR product is cleaved into 288 bp and 282 bp fragments **(**Fig. 1A**).**

### Rs3134069

The PCR products for T245G (rs3134069) (271 bp fragment) were amplified as described by Pitocco et al. [21], then they were digested with HinfI restriction endonuclease (Fermentas) and separated on 3% agarose gels. The 271 bp PCR product was cleaved into 202 bp and 69 bp fragments only in the presence of a T nucleotide at position 245 **(**Fig. 1B**).**

### BMD assessment

Lumbar (L2–L4) BMD (g/cm2) was performed on all patients using a Lunar DPX IQ-USA, software version 4.5. All scans were performed by the same technician, and evaluation was conducted by a single expert observer. The results of the BMD were compared with 352 healthy age- and sex-matched Egyptian controls [22, 23]and z-score was calculated. Patients with a BMD z-score <–2 was diagnosed as having low bone mass, whereas others were considered normal [24].

### Radiographic examination

Conventional film screen radiographs of all affected joints were obtained. The features evaluated as absent or present.

### Study power

The G*Power 3.1.9.7 was used to estimate the sample size using the following parameters; Effect size – 0.5, α err prob = 0.05, Power (1-β err prob) = 0.80, Allocation ratio N2/N1 = 0.7, Output: Non-centrality parameter δ = 2.8244066, Critical t = 1.9783804, Df = 130, Sample size group 1 = 78, Sample size group 2 = 54, Total sample size = 132, Actual power = 0.8005319. According to the The G*Power 3.1.9.7, the estimated sample size is 132 (78 cases and 54 controls). However, only 60 cases included as only available cases at our centre that fulfilled inclusion criteria while controls increased to 100 to enable more comparison between groups.

### Statistical analysis

Results were reported as means and medians for continuous or proportions for categoric variables. sRANKL and OPG levels and sRANKL/OPG ratio, OPG gene genotypes and alleles frequencies were compared between JIA patients and healthy controls. Genotypes and alleles frequencies, serum RANKL/OPG levels and other clinical features were compared between patients with BMD z-score below and above − 2 using univariate followed by multivariable linear regression analyses. All studied variables in univariate analyses at a *p* value < 0.05 were considered potential determinants and entered multivariable linear regression models. Hardy-Weinberg equilibrium was assessed with the Chi-square test or Fisher’s exact test, as appropriate. Statistical calculations were performed using Statistical Package for the Social Sciences (SPSS) Statistics Software, v.25.0 (SPSS Inc, Chicago, IL, USA), and a *p* value less than 0.05 was considered significant.

## Results

### Baseline clinical, laboratory and radiological characteristics of the patients

**Fig. 2:**
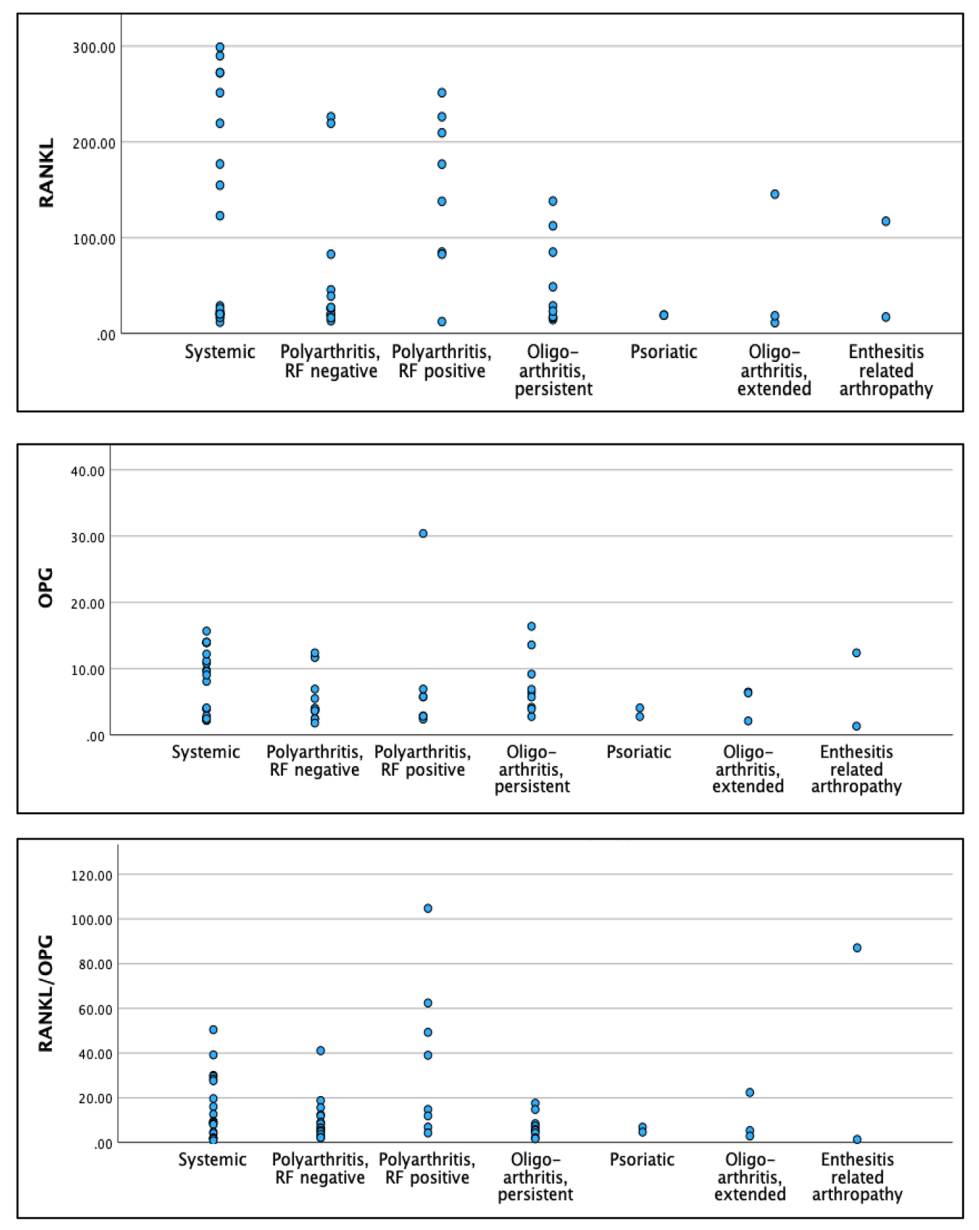
Scatter plots of RANKL, OPG and RANKL/OPG ratio in different patients’ groups

Sixty JIA patients were included in the study. Clinical, laboratory and radiological data of the patients are presented in Table 1. Systemic onset was the most frequent phenotype of the disease, rheumatoid factor (RF) was positive in 19 patients, narrow joint space was the most frequent radiological finding and methotrexate the most frequently used medication. Serum RANKL, RANKL/OPG ratio were significantly higher while serum OPG significantly lower in patients compared to controls. Serum RANKL, OPG and RANKL/OPG ratio in different patients’ phenotypes are presented as scatter plots in Fig. 2.

**Table 1:** Baseline clinical, laboratory and radiological characteristics of the patients

	Patients (N = 60) mean ± SD
**Age at diagnosis (years)**	7.7 ± 3.3
**Duration of illness (months)**	53.6 ± 34.2
**BMI (kg/m** ^**2**^ **)**	23.8 ± 1.9
**GFR (mL/min/1.73 m** ^**2**^ **)**	101.9 ± 5.9
**ESR (mm/h) (normal ≤ 10 mm/h)**	38.3 ± 30.9
**CRP (mg/L) (normal ≤ 10 mg/L)**	13.1 ± 10.98
**RF** (IgM) **(+ ve/-ve)**	19/41
**ANA (+ ve/-ve)**	5/55
**BMD z-score**	-0.7 ± 1.6
**Disease-onset type, n (%)**	
Systemic Oligo-arthritis, persistent Oligo-arthritis, extended Polyarthritis, RF negative Polyarthritis, RF positive Enthesitis related arthropathy Psoriatic arthritis	23(38.3%) 8 (13.3%) 3(5%) 14(23.3%) 8(13.3%) 2(3.3%) 2(3.3%)
**Number of tender joints**	3.2 ± 4.1
**Number of swollen joints**	2.4 ± 3.97
**Number of joints with restricted mobility**	1.5 ± 2.7
**Juvenile arthritis disease activity score** (JADAS) 27-joints	15.3 ± 12.7
**Juvenile arthritis damage index**:	
**Articular** **Extraarticular**:	8.01 ± 9.8 2.2 ± 2.1
**Medications: N(%)**	
**Corticosteroids** **NSAIDs** **Methotrexate** **Sulfasalazine** **Biological treatment**	41(68%) 35(58%) 48(80%) 2(3%) 12(20%)
**Cumulative steroid dose(mg/kg)**	46.2 ± 21.8
**Radiological findings: N(%)**	
**Soft tissue swelling** **Juxta-articular osteopenia** **Narrow joint spaces** **Erosions** **Deformities** **Periosteal reaction**	26(43%) 31(52%) 30(50%) 22(37%) 18(30%) 5(8%)

ESR: erythrocyte sedimentation rate, CRP: C-reactive protein, RF: rheumatoid factor, BMD: bone mineral density, NSAIDs: non-steroidal anti-inflammatory drugs, BMI: body mass index, GFR: glomerular filtration rate

### Genotypes and alleles distribution of the participants

The genotype frequencies of rs2073617 and rs3134069 of controls were within Hardy–Weinberg equilibrium (*p* = 0.9). OPG gene genotypes and alleles frequencies of the 2 studied SNPs did not show significant difference between patients and controls **(**Table 2**)** or between different disease phenotypes. Serum RANKL, OPG and RANKL/OPG ratio were significantly higher in patients with TT genotype of rs2073617 compared to other patients, while no significant difference in RANKL, OPG or RANKL/OPG between different genotypes of rs3134069 **(**Table 3**).** Serum RANKL, OPG and RANKL/OPG ratio distribution in control group is also presented in Table 3.

**Table 2:** Clinical and laboratory data of JIA patients and controls

	Patients (N = 60) mean ± SD	Controls (N = 100) mean ± SD	p-value
**Age at sampling (years)**	13(5–17)	**13(6–18)**	0.09
**Sex: Male/female**	22/38	**36/64**	0.9
**Height z-score**	-1.3(-2.4–1.5)	**0.5(-2.1–2.2)**	**< 0.001**
**BMI z-score**	0.8(-2–2.9)	**0.85(-0.9–2.7)**	0.06
**RANKL (ng/L)** **OPG (ng/L)** **RANKL/OPG**	85.8 ± 90.6 6.9 ± 5.2 16.1 ± 20.3	39.7 ± 58.3 11.7 ± 7.8 8.5 ± 22.1	**< 0.001** **< 0.001** **0.03**
**rs2073617 genotypes**:			0.14
**TT** **TC** **CC**	22(36.7%) 21(35%) 17(28.3%)	32(32%) 50(50%) 18(18%)	0.5 0.06 0.13
**rs2073617 Alleles**:			
**T** **C**	65(54.2%) 55(45.8%)	114(57%) 86(43%)	0.6
**rs3134069 genotypes**:			0.11
**TT** **TG** **GG**	12(20%) 30(50%) 18(30%)	34(34%) 36(36%) 30(30%)	0.06 0.08 1
**rs3134069 Alleles**:			
**T** **G**	54(45%) 66(55%)	104(52%) 96(48%)	0.23

BMI: body mass index, OPG: osteoprotegerin, RANKL: receptor activator of nuclear factor κB-ligand

**Table 3:** Serum RANKL, OPG and RANKL/OPG ratio in different genotypes and alleles of JIA patients

*JIA patients (N = 60)* median(min-max)							
	**rs2073617 genotypes**						***P***
	**TT**	**TC**		**CC**			
**Serum RANKL (ng/L)** **OPG (ng/L)** **RANKL/OPG**	104.7(27.7-298.9) 4.9(2.2–15.7) 18.2(4.1-104.75)	27.2(11.4-272.3) 5.8(1.8–30.4) 5.4(0.85–41.1)		23.2(13.4-272.3) 4.02(1.35–16.4) 6.9(1.4–87.1)			< 0.001*,0.1**, 0.03*** 0.8*,0.9**, 0.94*** 0.01*, 0.03**, 0.6***
	**rs3134069 genotypes**						
	**TT**	**TG**			**GG**		
**Serum RANKL (ng/L)** **OPG (ng/L)** **RANKL/OPG**	101.2(15.6-176.9) 3.8(1.35–13.9) 12.5(1.9–87.1)	42.8(11.4-298.9) 5.7(2.1–30.4) 8.8(0.85–104.8)			49.1(16.4-229.5) 5.6(1.8–14.1) 7.7(1.7–62.4)		0.7^, 0.8^^, 0.8^^^ 0.3^, 0.4^^, 0.8^^^ 0.3^, 0.8^^, 0.2^^^
Controls (N = 100) **median(min-max)**							
	**rs2073617 genotypes**						***P***
	**TT**		**TC**			**CC**	
**Serum RANKL (ng/L)** **OPG (ng/L)** **RANKL/OPG**	89.5(10.2–254) 3.6(1.4–14.8) 9.8(0.98–148)		4.05(1.1-138.8) 12.8(2.1–26.2) 0.48(0.05–28.2)			3.2(1.1–14.5) 13.3(4.8–54.1) 0.2(0.09–1.6)	< 0.001*,0.04**, < 0.001*** < 0.001*,<0.001**, 0.2*** < 0.001*, < 0.001**, < 0.001***
	**rs3134069 genotypes**						
	**TT**		**TG**			**GG**	
**Serum RANKL (ng/L)** **OPG (ng/L)** **RANKL/OPG**	89.5(4.3-254.1) 3.7(1.4–14.8) 9.7(0.34–148.1)		7.8(1-138.8) 13.2(2.1–26.2) 0.5(0.06–10.3)			2.5(1.2–14.5) 12.8(2.4–54.1) 0.2(0.05–1.6)	**< 0.001**^, 0.9^^, **< 0.001**^^^ **< 0.001**^, 0.002^^, 0.1^^^ **< 0.001**^, 0.1^^, **< 0.001**^^^

*:TT versus TC + CC, **: TC versus TT + CC, ***: CC versus TT + TC. ^: TT versus TG + GG, ^^: TG versus TT + GG, ^^^:GG versus TT + TG.

### Univariate and multivariate analysis of factors correlated to reduced BMD

Univariate analysis showed that rs2073617 TT genotype and T allele confer significant risk for developing low BMD in children with JIA. Serum RANKL, RANKL/OPG ratio, articular and extra articular damage index score were all significantly higher in patients with BMD z-score below − 2 compared to those with BMD z-score above − 2 and systemic-onset disease was more frequent in patients with BMD z-score < -2 **(**Table 4**).** Multivariate analysis showed rs2073617 TT genotype (p = 0.03, OR: 6.8, 95% CI:(3.1–18.2), RANKL/OPG ratio (*p* = 0.04, OR: 2.6,CI:(3.9-22.08), long disease duration (above 36 months) (*p* = 0.01, OR:5.2, 95%CI:(2.2–11.3) and the use of steroids (*p* = 0.01, OR: 4.5, 95%CI:(2.5–19.7) to be associated with decreased BMD in children with JIA.

**Table 4:** Clinical, laboratory and genetic comparison between patients with BMD Z-score above and below − 2 (Univariate analysis)

	Patients with BMD z-score <-2 (N = 19) N (%)	Patients with BMD z-score >-2 (N = 41) N (%)	*P*	*OR (95% CI)*
**T950C (rs2073617)**			**< 0.001**	
**Genotypes**:				
**TT** **TC** **CC**	14(73.7%) 3(15.8%) 2(10.5%)	8(19.5%) 18(43.9%) 15(36.5%)	**< 0.001** **0.04** 0.05	11.5(3.2–41.6) 0.2(0.06–0.95) 0.2(0.04–1.007)
**Alleles**:				
**T allele** **C allele**	31(81.6%) 7(18.4%)	34(41.5%) 48(58.5%)	**< 0.001**	6.2(2.5–15.9)
**T245G (rs3134069)**			**0**.96	
**Genotypes**:				
**TT** **TG** **GG**	4(21%) 9(47.4%) 6(31.6%)	8(19.5%) 21(51.2%) 12(29.3%)	0.89 0.78 0.86	1.1(0.29–4.2) 0.86(0.29–2.5) 1.1(0.34–3.6)
**Alleles**:				
**T allele** **G allele**	17(44.7%) 21(55.3%)	37(45.1%) 45(54.9%)	0.97	0.96(0.45–2.1)
**RANKL (ng/L)** **OPG (ng/L)** **RANKL/OPG**	129.2 ± 101.4 5.4 ± 3.6 27.7 ± 25.7	65.7 ± 78.6 7.6 ± 5.8 10.6 ± 14.7	**0.01** 0.12 **0.002**	(15.5-111.4) (-5.1-0.6) (4.03–30.2)
**Sex male/female N(%)**	3(15.8%)/16(84.2%)	19(64.3%)/22(53.7%)	**0.02**	0.22(0.06–0.86)
**Age at diagnosis/years**	8.5 ± 3.3	7.3 **± 3.2**	0.2	(-0.7- 2.9)
**Duration of illness (months)** **Above/below 36 months N(%)**	64.4 ± 32.1 16(84.2%)/3(15.8%)	48.6 ± 34.4 20(48.8%)/21(51.2%)	0.09 0.009	**8.8**(-2.9- 34.5) 6.7(1.1–12.9)
**BMI Z-score**	1.1 ± 1.01	0.79 ± 1.34	0.37	0.2(-1–0.38)
**Type of onset: n (%)**				
**Systemic** **Polyarticular** **Oligoarticular** **Psoriatic** **Enthesitis-related arthropathy**	11(52.6%) 6(31.6%) 2(15.8%) 0 0	12(29.3%) 16(39%) 9(22.1%) 2(4.8%) 2(4.8%)	0.03* 0.57** 0.29***	2.7(0.87–8.2) 0.7(0.2–2.3) 0.7(0.16–2.8)
**Steroid use yes/no, N(%)** **Methotrexate use yes/no N(%)** **Biological treatment yes/no, N(%)**	17(89.5%)/2(10.5%) 16(84.2%)/3(15.8%) 5(26.3%)/14(73.7%)	24(58.5%)/17(41.5%) 32(78%)/9(22%) 7 (17.1%)/34(82.9%)	**0.02** 0.58 0.41	6.02(1.2–29.6) 1.5(0.35–6.3) 1.7(0.47–6.4)
**Cumulative steroid dose (mg/kg)**	75.7 ± 24.1	38.1 ± 16.1	**0.003**	5.6(6.9-32.25)
**Number of swollen joints**	3.3 ± 4.8	2.02 ± 3.5	0.3	1.3(-0.96-3.4)
**Number of tender joints**	4.3 ± 4.6	2.7 ± 3.7	0.15	0.9(1.6–1.2)
**Number of joints with limited mobility**	2.05 ± 3.3	1.2 ± 2.4	0.3	0.3(-0.72-2.3)
**Rheumatoid factor + ve/-ve** **N(%)**	6(31.6%)/13(68.4%)	13(31.7%)/28(68.3%)	0.9	1.1(0.4–3.3)
**JADAS-27**	14.9 ± 13.5	15.4 ± 12.4	0.9	1.8(-7.8 -6.9)
**Damage index**				
**Articular** **Extraarticular**	12.8 ± 10.5 3.2 ± 2.3	5.7 ± 8.7 1.8 ± 1.7	**0.008** **0.009**	0.8(1.9–12.2) 1.3(0.4–2.5)

-*: Systemic type versus all other types, ** : polyarticular type versus others, ***: oligoarticular type versus others

BMD: bone mineral density, JADAS-27: Juvenile arthritis disease activity score, OPG: osteoprotegerin, RANKL: receptor activator of nuclear factor κB-ligand, BMI: Body mass index

### Other clinical correlations

Serum OPG significantly correlated to composite disease activity (JADAS-27) (r = 0.3, *p* = 0.02), RANKL/OPG ratio was significantly higher in patients versus those without bony erosions (26.3 ± 27.6 vs. 10.1 ± 11.3, *p* = 0.002). Cumulative steroid dose showed negative significant correlation to BMD (*p* = 0.01, r=-0.38). No significant difference in RANKL/OPG ratio was spotted between different disease phenotypes. Also, no significant correlation reported between RANKL/OPG and articular (*p* = 0.09, r = 0.2) and extraarticular damage scores (*p* = 0.2, r = 0.2).

Given the distinct pathophysiology and differences in treatment exposures (use of steroids, different biologics, etc.), a supplementary table (Supplementary Table 1) was added detailing the systemic JIA patients’ data, genotypes and BMD status classified into good disease control and active disease based on cut-off points of JADAS-27 as defined by Consolaro et al., 2016 [25].

## Discussion

Globally, nearly 3 million children and young adults are suffering from JIA. The region of Africa and Middle East constitute a diverse group of ethnicities, socioeconomic conditions, and climates which influence the prevalence of JIA [26] which is noted to be lower than the range of the global estimate (3.8–400 per 100,000) [27]. Consistent with previous reports from Africa and around the world, female predominance was observed in the current work, but higher age of onset was reported [28–30]. Oligoarticular type is reported to be the most frequent followed by polyarticular and systemic phenotypes in different studies [31, 32] which is against our current report in which systemic phenotypes was the most prevalent (38%) while oligoarticular type was reported only in 18% of the cases. Uveitis was not reported in our current cohort. This is consistent with two studies from Oman which did not report any cases of uveitis from their cohorts [33, 34] and with Saurenman et al., who reported a lower risk of uveitis in Arab and Asian JIA patients compared to European or North American ethnicities [35].

Reduced BMD in children with JIA have been replicated in many studies and correlated to wide range of clinical, laboratory and radiological parameters of the disease [36, 37] with the lowest BMD Z-scores reported to be in systemic onset, polyarticular involvement, high steroid doses, and longer disease duration [38, 39]. Low BMD was also reported in adults with history of JIA [40]. In the current work low BMD was reported in 31% of the patients which is against a recent report by Charuvanij et al., who reported no low lumbar BMD and only 5% low total BMD in their cohort of 38 JIA subjects [41] while El Badri et al., reported low BMD in 50% of the studied Moroccan children with JIA [42]. This difference may be related to different sample size, disease duration, characteristics of included subjects and definition of low BMD or osteoporosis.

A high RANKL/OPG ratio in patients compared with controls has been reported in children with nephrotic syndrome [3], children with type 1 diabetes milletus,[43] and juvenile SLE [8] whereas Ozkaya et al. found an increased OPG/RANKL ratio in children with CKD representing a compensatory mechanism to the negative balance of bone remodeling in this disease [44]. However, the role of RANKL/OPG homeostasis in BMD in JIA children explored in few studies [45–51] with OPG gene polymorphism in children with JIA studied once [47] [to the best of our knowledge]. Main findings of the current study and previous literature on RANKL-OPG axis and OPG gene polymorphism in JIA patients are presented in Table 5.

**Table 5:** **Main findings of the current study and previous literature on RANKL-OPG axis and OPG gene polymorphism in JIA.**

Author [ref]	Year	Patients /Control	Country	Patients’ age (years)	Main findings
**Eid et al.; [current]**	2022	60/100	Egypt	12.05 ± 3.2	Egyptian children with JIA have decreased BMD. rs2073617 TT genotype and T allele, RANKL/OPG ratio are possible predictor for reduced BMD in JIA
**Shalaby et al.;**[51]	2021	40/40	Egypt	11.14	High RANKL and low OPG levels appear to be associated with low bone mass in JIA patients.
**Alkady et al.**, [6]	2011	70/30	Egypt	11.8 ± 3.37	RANKL was higher, OPG and OPG/RANKL ratio were significantly lower in patients. OPG/RANKL ratio is correlated with most clinical characteristics and disease activity variables.
**Lien et al.**, [1]	2010	90/90	Norway	10.1 ± 3.2	RANKL level was higher, whereas OPG and OPG/ RANKL levels were lower in patients. Patients who received steroids or antirheumatic drugs at follow-up had higher OPG/RANKL ratio.
**Agrawal et al.;** [50]	2009	41 ERA 4 Poly JIA	India		Serum RANKL was detectable in 251 ERA patients, 4 Poly JIA patients. Median synovial fluid RANKL level in patients with ERA was higher as compared to osteoarthritis and poly JIA but was comparable to RA.
**Spelling et al.** [45]	2008	30/30	Brazil	11.07 ± 3.77	Patients with active disease and bone erosions had higher serum levels of RANKL and a lower OPG/ RANKL ratio than controls and patients without bone erosions.
**Sarma** **et al.;** [48]	2008	70/30	India	5–32	RANKL, OPG were elevated in JIA patients as compared to controls. There was no difference in levels among different types of JIA RANKL/OPG ratio was elevated in all subtypes of JIA.
**Buzi et al.**,[46]	2004	19/64	Italy	8.5 ± 1.74	OPG higher in patients while RANKL levels did not differ between patients and controls.
**Masi et al.;** [47]	2004	84/40	Italy	5.6–11.4	OPG/RANKL ratio in patients was higher than in controls. A significant difference in serum OPG levels was found between patients with and without erosions. Subjects with CC genotype had a higher lumbar spine BMD.
**Varsani et al.** [49]	**2003**	**18/10**	United Kingdom	**-----**	mRNA for RANKL was detectable in the T-cell fraction from JIA patients but not in that from controls.

OPG: osteoprotegerin, RANKL: receptor activator of nuclear factor κB-ligand, JIA: juvenile idiopathic arthritis, ERA: enthesitis-related arthritis, BMD: bone mineral density, RA: rheumatoid arthritis

Decreased BMD in JIA is multifactorial. Lien et al.; reported that JIA patients with oligo- or polyarthritis phenotypes had significantly lower levels of OPG early in the disease course compared to controls and that baseline RANKL was a significant negative predictor of total body lean mass [1]. These findings are consistent with Geusens et al.; findings in newly diagnosed RA patients as the first year OPG:RANKL ratio, as measured at baseline, independently predicted 5-year radiographic progression of joint damage as progression of radiographic damage was greatest in patients with a low OPG:RANKL ratio and was lowest in patients with a high OPG:RANKL ratio. [52]. Shunle et al. studied 45 recent-onset untreated SLE patients and reported that the expressions of the RANKL and OPG genes were significantly reduced in initial SLE compared with normal controls and that initial SLE patients had significantly lower BMD compared to controls [53]. This explains the importance of concomitantly studying serum levels of RANKL/OPG and OPG gene polymorphisms for better recognize bone loss and the clinical significance of the balance between OPG and RANKL in children with JIA.

The correlation of steroids to BMD in different disorders and even between different studies on the same disease is controversial. In the current work we reported a negative significant correlation between cumulative steroid dose and BMD. Sušić et al., reported similar association to cumulative steroid dose in their cohort of 75 children with JIA [36] as well as El Badri et al., [42]. However, Zhizho et al.; reported no significant correlation between cumulative dose of steroids and BMD [54]. Methotrexate has been consistently associated with reduced BMD in children treated for childhood cancer [55], however in the current work the use of methotrexate did not correlate to BMD, which is consistent to previous published reports [42]. This difference may be related to smaller dose of methotrexate used in rheumatic diseases compared to malignancies.

In the current work, no difference reported in BMD in those who received versus those who did not receive biological treatment. Biological treatment with infliximab and etanercept in children with JIA is associated with decrease in disease activity [56]. The positive influence of treatment with TNF*α* antibodies was also documented upon the skeleton. Simonini was the first to demostrate increased bone mass after 1-year etanercept treatment in children with JIA; reduction of bone loss was associated with therapeutic response with decreased disease activity [57]. Etanercept also improves the linear growth in children with JIA [58].

Based on the concept that increased local or systemic RANKL/OPG may favour increased osteoclastic bone resorption in different metabolic bone disorders, exogenous injection of recombinant OPG has been efficaciously used in various animal models of bone diseases to modulate bone and mineral abnormalities caused by inadequate osteoclast activity [11]. OPG administration exerted a brisk and marked effect and had no adverse effects [59, 60]. A controlled clinical study has confirmed the antiresorptive effect of recombinant human OPG therapy in postmenopausal women as monthly injection of OPG significantly decreased the concentrations of deoxypyridinoline indicating that the data obtained in animals could be replicated in humans [61].

### Limitations and strength

The current study was limited by the cross-sectional design, the sample-size that hinders determining a cause–effect relationship between the studied OPG gene SNPs and the risk of osteoporosis in children with JIA and not assessing synovial RANKL levels. However, this study is the first to explore the potential role of the OPG gene rs2073617 and rs3134069 polymorphisms with concomitant evaluation of serum RANKL/OPG levels in susceptibility to reduced BMD in Egyptian children with JIA.

## Conclusions

Egyptian children with JIA have decreased BMD. rs2073617 TT genotype and T allele, RANKL/OPG ratio are associated with reduced BMD in patients with JIA. Our results underline the importance of frequent monitoring of BMD in JIA children and trying to control disease activity to preserve long term bone health.

## Electronic supplementary material

Below is the link to the electronic supplementary material.

Supplementary Material 1

Supplementary Material 2

## Data Availability

Relevant, de-identified data can be made available on request.

## Abbreviations

ANA: Anti-nuclear antibodies
BMD: Bone mineral density
BMI: Body mass index
CRP: C-reactive protein
DEXA: Dual energy X-ray absorptiometry
ERA: Enthesitis-related arthropathy
ESR: Erythrocyte sedimentation rate
ILAR: International League of Associations for Rheumatology
JADAS: Juvenile arthritis disease activity score
JADI: Juvenile arthritis damage index
JIA: Juvenile idiopathic arthritis
OPG: Osteoprotegerin
PCR-RFLP: Polymerase chain reaction - restriction fragment length polymorphism
RANKL: Receptor activator of nuclear factor κB-ligand
RA: Rheumatoid arthritis
RF: Rheumatoid factor
SNP: Single nucleotide polymorphism
SLE: Systemic lupus erythematosus
TNF: Tumour necrosis factor
