# Supplementary material for: Bone mineral density in egyptian children with juvenile idiopathic arthritis: possible correlation to serum RANKL / osteoprotegerin (OPG) ratio and OPG gene polymorphisms
Source: Pediatr Rheumatol Online J. 2023 Jun 16;21:58. doi: 10.1186/s12969-023-00843-6 (PMC10273548; doi:10.1186/s12969-023-00843-6)
Supplement: Supplementary file 2 — Supplementary Material 2 [file 12969_2023_843_MOESM2_ESM.docx]

**Supplementary** **Table 1: Baseline clinical, laboratory and radiological characteristics of systemic JIA patients**

|  | **Systemic JIA patients**  **(N=23)** |
| --- | --- |
| **Age at diagnosis (years)** mean± SD | 7.33±3.2 |
| **Duration of illness (months)** mean± SD | 60.6±35.9 |
| **BMD z-score ,** mean± SD | -0.78±1.7 |
| **Juvenile arthritis disease activity score** (JADAS) 27-joints , mean± SD | 11.2±10.2 (0-28) |
| **JADAS-27 classification (%)**  Inactive diseases: ≤1  Mild activity: 1.1-3.8  Moderate activity: 3.9-8.5  High activity: > 8.5 | 6(26.1%)  1(4.3%)  6(26.1%)  10(43.5%) |
| **Juvenile arthritis damage index:** mean± SD  **Articular**  **Extraarticular:** | 8.7±10.3  2.9±2.1 |
| **RANKL (ng/L)**  **OPG (ng/L)**  **RANKL/OPG** | 103.1± 110.1  7.6± 4.8  14.6±13.5 |
| **rs2073617 genotypes:**  **TT**  **TC**  **CC**  **rs2073617 Alleles:**  **T**  **C** | 11(47.9)  5(21.7%)  7(30.4%)  27(58.7%)  19(41.3%) |
| **rs3134069 genotypes:**  **TT**  **TG**  **GG**  **rs3134069 Alleles:**  **T**  **G** | 6(26.1%)  10(43.5%)  7(30.4%)  22(47.8%)  24(52.2%) |

JIA: Juvenile idiopathic arthritis, OPG: osteoprotegerin, RANKL: receptor activator of nuclear factor κB-ligand, BMD: bone mineral density, JADAS: Juvenile arthritis disease activity score
