# Supplementary material for: Bone mineral density in egyptian children with juvenile idiopathic arthritis: possible correlation to serum RANKL / osteoprotegerin (OPG) ratio and OPG gene polymorphisms
Source: Pediatr Rheumatol Online J. 2023 Jun 16;21:58. doi: 10.1186/s12969-023-00843-6 (PMC10273548; doi:10.1186/s12969-023-00843-6)
Supplement: Supplementary file 1 — Supplementary Material 1 [file 12969_2023_843_MOESM1_ESM.docx]

**Response to reviewer’s Comments**

**To editor:** Thanks a lot for considering our work for evaluation and possible publication in **“Pediatric Rheumatology Journal”.**

Please find below responses to reviewers’ comments point by point and the required changes were done to the manuscript highlighted in red.

**To reviewers:** Thanks a lot for your careful revision of our work and valuable comments. We have tried our best to address all your comments. Please find responses below and the required modifications were done to the manuscript highlighted in red.

**Reviewer 1:**

| **Comment** | **Response** |
| --- | --- |
| **Major comments:** | |
| 1. The authors have conducted similar studies on childhood SLE. (Eid R, Abdelsalam M, Fathy AA, et al. Predictors of decreased bone mineral density in childhood systemic lupus erythematosus: possible role of J Pediatr Endocrinol Metab. 2021;35(1):79-87. Published 2021 Nov 18. doi:10.1515 2. \/jpem-2021-0496) As the SNP results for the control children are the same, thus the authors appear to analyze the same 100 control children. However, the two articles' background factors (e.g., age) of these control children are different. The authors need to list their paper as a reference and explain whether the control children are the same or different. | Thanks a lot for this critical observation.  Yes, the 100 control children in the current manuscript are the same control group included in the mentioned SLE study.  We apologize for this typing error as we used an old table templet of another JIA article for writing and did not change the background data.  The correct background data were added to the manuscript, Table 2 and our previous SLE study was listed as a reference. |
| 3. The authors should show whether the subjects receive vitamin D, calcium, and therapies for osteoporosis, including bisphosphonates, and should include them in the analysis. | None of the included 60 patients received bisphosphonates up to the time of sampling and BMD assessment in the study. Unfortunately, data regarding calcium and vitamin D consumption were not included in the analysis because of patients’ inconstant interrupted undocumented doses and intake durations.  This was added to the manuscript, methods section. |
| 4. The authors should show OR and 95%CI for valuables which they found a significant association with decreased BMD in the multivariable analysis. | OR and 95%CI for valuables which found to be of significant association with decreased BMD in the multivariable analysis were added to results section , page 10, line 267-269. |
| **Minor comments:** | |
| 1. In Table 4, "OR" is missing after the row "Age at diagnosis/years." | The missing OR after the row "Age at diagnosis/years." were added to table 4. |

**Reviewer 2:**

| **Comment** | **Response** |
| --- | --- |
| 1. What is the normal range for ESR and CRP in this institution. This needs to be added to table 1. | In our institution:  Normal values for ESR ≤ 10 mm/hr  Normal values for CRP ≤ 10mg/L    This was added to Table 1 |
| 2. The analysis of cumulative steroid dose and serum RANKL / Osteoprotegerin (OPG) ratio and OPG gene polymorphism is not convincing. Would it be possible to compare subgroups in systemic JIA with good disease control to group with active disease. Compare patients on biologics to patients not on biologics. compare patients on steroids to patients not on steroids. It might be the numbers are too small to be able to do this. Further discussion needed if further analysis of subgroups not possible. | Wallace and coworkers in 2004 [reference 16] developed the preliminary criteria for definition of disease remission in JIA which was later on modified in 2011[reference 17]. When the criteria for inactive disease are met for a minimum of six consecutive months while the patient is receiving anti-rheumatic medications, the patient is classified as being in the state of clinical remission with medication.  When the criteria for inactive disease are met for a minimum of 12 consecutive months after the patient has discontinued all anti-rheumatic medications, the patient is classified as being in the state of clinical remission without medication.  However, as we did not follow up our included patients (being a cross sectional study) for the required period to define clinical status we did not include this clinical assessment for classifying our patients into active and inactive diseases. This was added to methods section highlighted in red.  The other way for assessment of disease activity is using one of the disease activity scores. In the current work we used JADAS-27 and the values of the score were compared between patients with BMD z-score above and below -2 (Table 4 , highlighted in red).  A supplementary table was added detailing the systemic JIA patients data, genotypes and BMD status classified into good disease control and active disease based on cut-off points of JADAS-27 as defined by Consolaro et al., 2016[ reference 25]  As JADAS score was assessed only once at time of the study, which doesn’t provide a full picture of disease status, control and response to medications, we used damage index(articular and extraarticular) to evaluate the long term effects of the disease which was also correlated to BMD status shown in Table 4.  The comparison of BMD between patients on steroids and those not on steroids is presented in Table 4 (highlighted in red).  The comparison of BMD between patients on biologics and those not on biologics is presented in Table 4 (highlighted in red).  A brief discussion on relation of biological treatment of JIA on BMD was added to “Discussion” section, page 13, lines 336-342 highlighted in red. |

**Reviewer 3:**

| **Comment** | **Response** |
| --- | --- |
| **Introduction**   - It would be helpful for context to include some background information about why these particular SNPs were selected for analysis. | The required modifications added to the introduction section. |
| **Methods**   - Question the utility of lumping systemic JIA together with other forms of JIA given the distinct pathophysiology and differences in treatment exposures (use of steroids, different biologics, etc. - It seems it would be important to assess vitamin D status in a study focused on susceptibility to low BMD, but there does not appear to be any mention of vitamin D. | - Due to small number of patients in our institution with systemic JIA (23 patients) and for comparative purposes with other studies which included systemic JIA along with other types. A supplementary table was added detailing the systemic JIA patients’ data, genotypes, and BMD status.   -Unfortunately, Data about calcium and vitamin D consumption were not included in the analysis because of patients’ inconstant interrupted undocumented doses and intake durations.  This was added to the manuscript, methods’ section. |
| **Results**   - Were the distributions of RANKL, OPG and RANKL/OPG levels assessed? Based on the large standard deviations presented in tables 3 and 4, it appears they are unlikely to be normally distributed – as such, reporting and statistical comparison of medians/IQR would be more appropriate than means/SD. On a related note, for transparency, would recommend graphically representing the raw data and distribution (e.g., dot plots by group) in a supplemental figure. - The estimates from the multivariable analysis should be reported somewhere either in the text or a table. Listing only the p-values is unhelpful for interpretation. - The authors show a relationship between genotype and serum RANKL and RANKL/OPG in the JIA patients – but is this also true of controls without JIA? This would be important for interpretation, as these findings could be applicable across the general population and not unique to JIA. | Thanks a lot for this important observation.  The presentation and analysis of RANKL, OPG, RANKL OPG ration levels was modified as recommended  A figure was added showing scatter plot for serum RANKL, OPG and RANKL/OPG ratio in different patient groups.  -OR and 95%CI for valuables which found to be a significant association with decreased BMD in the multivariable analysis were added to results’ section, page 10, line 267-269.  The relationship between genotype and serum RANKL and RANKL/OPG in controls was presented in Table 3 |
| **Table 4**   - Somewhere in the title or legend it should be indicated that the estimates are from univariable/unadjusted regression models - Recommend presenting all binary variables (e.g., Steroid use) as n (%) rather than n1/n2 to be consistent throughout the table - Is cumulative steroid dose presented in prednisone equivalents? | The recommended modifications done to table 4.  The recommended changes done to Table 4.  Yes. |
| **Minor**   - It appears that the incorrect table number is referenced on line 308 - Please ensure all significant figures are consistent within each table - As this was a cross-sectional study, would avoid use of the term “predictor” in reference to factors associated with BMD other than genotype. | The recommended modifications and corrections have been done.  -the recommended corrections were done all through the manuscript. |

**Reviewer 4:**

| **Comments** | **Response** |
| --- | --- |
| Two things in the manuscript have to be corrected. Line 145 should be ligand not legend. Line 246 states 19 patients were RF positive, but Table 1 states only 8 were RF positive. This needs to be corrected.  Overall, the data is well presented, but may not be comparable in American/Northern European JIA populations in lieu of the different distributions of onset types. More correlations with onset type could have possibly defined this better than just the correlations with different genotypes and alleles. Overall, the manuscript does demonstrate that genotype and allele with RANKL/OPG ratios may be a predictor of reduced BMD in JIA and could be a measure in the future. | The recommended modifications and corrections have been done.  Thanks a lot for your comment on data presentation.  More detailed analysis of clinical data, genotypes, BMD of the systemic JIA onset-type being the most frequent in the current study is presented in Supplementary table 1. |

**Reviewer 5:**

| **Comments** | **Response** |
| --- | --- |
| **Major comments:** | |
| 1.      Method section and Table 1:  If the authors evaluated JADAS-27 score at the time of sampling, all patients with JIA who suffered from moderate to severe disease activities are assumed to have high scores. Therefore, evaluating the JADAS-27 score at sampling induces to the results of high serum RANKL and RANKL/OPG ratio. The authors should describe when they evaluated the JADAS-27 score (at diagnosis or at the time of collecting samples).  2.      Table 3: It is well known that patients with systemic JIA receive a large amount of glucocorticoid to achieve response rather than other types of JIA. Glucocorticoid is also known to suppress OPG expression, therefore, the authors should separate systemic JIA and others for evaluation of serum RANKL/OPG ratio and OPG gene genotypes. Otherwise, the authors mention the cumulative dose of glucocorticoid in each type of JIA. | 1. JADAS-27 was evaluated at time of sampling. This was added to methods section. 2. A supplementary table detailing data and laboratory, BMD results of systemic JIA group was added (Supplementary Table 1) |
| **Minor comments:** 1.      The authors should add the description regarding Table 5 in the text. 2.      L254: Extra 'rs' should be deleted. | 1. The description was added to discussion section. 2. Extra 'rs' should be deleted was deleted. |

**Reviewer 6:**

| **Comments** | **Response** |
| --- | --- |
| Interesting and well-written article with a correct methodology. I wonder why the author chose Lumber BMD instead of whole-body BMD | Thanks a lot for your comment.  We chose lumbar BMD instead of whole-body scan:   1. The data Our normal reference control group of 352 healthy age- and sex-matched Egyptian controls which was used to calculate z-score for our patients includes only lumbar BMD normal reference values [references 22,23] 2. None of our patients received sedation to perform BMD assessment so, we preferred lumbar assessment as it requires much less time than whole body scan. |
